# Mapping strategies, components, and theories used in health education and physical activity interventions to prevent cardiovascular diseases in adults living with HIV: A scoping review protocol

**DOI:** 10.1371/journal.pone.0312969

**Published:** 2025-08-01

**Authors:** Micah Mutuna Simpamba, Brian Chanda Chiluba, Yvonne Colgrove, Mercy Wamunyima, Esther Munalula-Nkandu

**Affiliations:** 1 School of Health Sciences, University of Zambia, Lusaka, Zambia; 2 Department of Physical Therapy, Rehabilitation Science and Athletic Training, University of Kansas Medical Center, Kansas City, Kansas, United States of America; 3 Medical Library, University of Zambia, Lusaka, Zambia; Xiamen University - Malaysia Campus: Xiamen University - Malaysia, MALAYSIA

## Abstract

**Introduction:**

Cardiovascular diseases (CVDs) have become the most significant contributor to non-HIV/AIDS-related deaths in people living with HIV (PLWH). The increased risk for CVDs in PLWH is attributed to a combination of traditional risk factors, the effects of chronic inflammation, and antiretroviral therapy-related metabolic changes.

**Objective:**

The main objective of this scoping review is to systematically map and summarize evidence on the strategies, components, and theories used in health education and physical activity interventions to prevent cardiovascular diseases in PLWH.

**Inclusion criteria:**

Peer-reviewed publications including relevant grey literature published in English will be included. The literature will focus on health education and physical activity interventions for people living with HIV, at risk of CVDs, aged between 18 years to 65 years. The literature publication will range between 2000 and June, 30th 2025, and intervention studies will include diverse research designs and literature sources from a broad array of settings including healthcare and community settings.

**Methods:**

Electronic databases to be searched will include PubMed, Scopus, CINAHL, and Embase, as well as grey literature such as Google Scholar, WHO Digital Publications (IRIS), and Proquest Thesis and Dissertation Global. A three-step process of screening consisting of titles/abstracts screening, full article screening, and manual search for references from identified articles will be conducted by two independent reviewers. In case of any discrepancies or disagreements, a third reviewer will be called upon to address the controversy. A data extraction form will be adapted from the Joanne Briggs Institute (JBI) standardized data extraction tool and key findings will be presented in tabular or diagram format followed by a narrative summary. Reporting of the scoping review will follow the Preferred Reporting Items for Systematic Reviews and Meta-Analyses extension for Scoping Reviews (PRISMA-ScR) checklist.

**Review protocol registration:**

The scoping review protocol is registered with Open Science Framework (https://osf.io/4wfkh/).

## Introduction

HIV/AIDS is a global public health concern that affects millions of people worldwide, with an estimated 26.6 million HIV-positive people living in the African region [1]. With the successful scale-up of antiretroviral therapy, HIV-related morbidity, and mortality have reduced and the attention on health care has now shifted to encompass the growing prevalence of non-communicable co-morbidities [2]. Cardiovascular diseases (CVDs) have become the most significant contributor to non-HIV/AIDS-related deaths in PLWH in both high-income countries and low and middle-income countries (LMICs) [3,4]. People living with HIV experience an elevated risk of cardiovascular complications, often attributed to a combination of traditional risk factors, the effects of chronic inflammation, and antiretroviral therapy-related metabolic changes [5]. The prevalence of CVDs and risk factors among PLWH are reported to be high in LMICs [6–8]. According to a systematic review that was done in LMICs, CVD risk factors that were reported in PLWH include elevated low-density lipoprotein 23.2% (95% CI 15.2–33.6), hypercholesterolemia 22.2% (95% CI 14.7–32.1), hypertension 21.2% (95% CI 16.3–27.1), low high-density lipoprotein 52.3% (95% CI 35.6–62.8), and obesity 7.8% (95% CI 4.3–13.9) [8]. Despite the high prevalence of CVDs among PLWH, many LMICs have not successfully implemented programs to address these non-communicable diseases (NCDs) in this population.

Given the high prevalence of traditional CVD risk factors in the HIV-positive population, adopting a healthy lifestyle should be the first step towards achieving primary and secondary prevention of CVDs [9]. Promoting a healthy lifestyle focusing on exercise, a healthy diet, and a healthy body weight forms the foundation of the clinical management of CVD risk among PLWH [10]. Hence, in response to this emerging health concern, health education and physical interventions can be integral components of preventive strategies aimed at reducing the incidence and impact of CVDs in the HIV-positive population.

Health education is the process of communication to provide knowledge and life skills related to health that are beneficial to the health of the individual as well as the community, including information on the underlying social, economic, and environmental factors that impact health [11]). Furthermore, health education provides the motivation, skills, and conviction needed to take the initiative to enhance behavioural and lifestyle aspects of health [11]. Health education interventions have shown promising results in enhancing cardiovascular risk profiles, including blood pressure, lipid profile, and cardiovascular risk score in various populations [12]. The benefits of health education interventions were also reported in a randomized controlled study of education and a home-based pedometer walking program that was carried out at an outpatient clinic in Johannesburg, South Africa, on HIV-positive individuals with risk factors for ischemic heart disease [13]. In this study, it was revealed that the health education and home-based pedometer walking program improves physical activity levels and has beneficial effects against ischemic heart disease risk factors [13].

Likewise, physical activity interventions, ranging from exercise regimens to lifestyle modifications, hold promise in mitigating cardiovascular risk factors and improving overall cardiovascular health. Engaging in regular physical activities, such as brisk walking, jogging, cycling, or swimming has several benefits on the cardiovascular system, including improved blood circulation, and efficient heart function, as well as addressing various risk factors associated with CVDs [14]. The advantages of physical activity interventions in PLWH have become more widely recognized as effective strategies for preventing CVDs. In a systematic review that examined the safety and effectiveness of aerobic exercise interventions in adults living with HIV, it was reported that these exercises are safe and beneficial for this population so long they are medically stable [15]. O’Brien and colleagues [15] noted that engaging in aerobic exercise or a combination of aerobic and resistive exercise among adults with HIV at least three times per week for five weeks can enhance their quality of life and cardiorespiratory fitness. Additionally, regular exercise improves lipid profiles by reducing levels of harmful LDL cholesterol, and increasing levels of beneficial HDL cholesterol, which can help prevent atherosclerosis and reduce the risk of CVDs [14].

This scoping review aims to systematically explore various strategies, components, and theories employed in physical activity and health education interventions specifically tailored for PLWH. The review will synthesize the existing literature to bring clarity to the current state of knowledge on the preventative strategies utilized to address cardiovascular risks in this specific group. Considering the unique challenges faced by individuals living with HIV, coupled with the increased risk for CVDs, this scoping review will offer a systematic and exploratory examination of the available evidence on CVD risk preventive measures. By exploring the diverse intervention strategies, components, and theories used in preventive interventions, this scoping review will contribute valuable information to inform evidence-based practices, refine intervention strategies, and direct future research initiatives.

A scoping review is preferred to a systematic review because it is the most appropriate type of literature review that will address a broad research question [16], such as ours which looks at strategies, components, and theories used in health education and physical activity interventions for PLWH. In addition, a scoping review will enable us to identify knowledge gaps, produce background evidence on the phenomenon under investigation, and clarify concepts and definitions related to the research [17]. This will enable the identification of trends and best practices in addressing cardiovascular health in PLWH, and ultimately inform the development of effective interventions and guiding future research on this topic. From this author’s perspective, the evidence which will be generated from this scoping review will contribute to the design and adaptation of the proposed Physiotherapy-led health education and physical activity program in the clinical setting in two (2) healthcare facilities situated in low-resource areas.

The research objectives and questions for this scoping review is constructed using the population, concept and context elements. The population will be people living with HIV, the concept will be health education and physical activity interventions and the context will include a broad array of settings across the globe.

### General objective

The general objective of this scoping review is to systematically map and summarize the existing literature on strategies, components, and theories utilized in health education and physical activity interventions aimed at preventing CVDs in adults living with HIV in a range of settings where these interventions are implemented.

### General review question

What strategies, components, and theories are reported in settings where health education and physical activity interventions are implemented to prevent CVD in people living with HIV?

### Primary review questions

#### Theme 1: Heath education strategies and components.

1. What health education strategies and components have been used in various settings to prevent cardiovascular diseases in people living with HIV?

#### Theme 2: Physical activity strategies and components.

1. What strategies and components have been used in different settings to implement physical activity interventions for prevention of CVDs in PLHIV?

#### Theme 3: Theoretical underpinnings of health education and physical activity interventions.

1. What theories or theoretical frameworks have guided the development and implementation of health education and physical activity interventions for cardiovascular disease prevention in people living with HIV?

### Secondary review questions

1. What evidence exists to support the effectiveness of health education and physical activity interventions in improving cardiovascular health among adults living with HIV?
2. What are the existing gaps or limitations in current CVD prevention interventions for PLWH, particularly in LMIC contexts?

## Inclusion criteria

The elements of a scoping review that are essential to determining the inclusion criteria are the ‘PCC’ mnemonic, which refers to the Population, Context, and Concept [18].

### Population

Studies will be eligible if the study population includes both male and female HIV-positive patients on ART aged between 18 years to 65 years, living in both High-income and low and middle income countries, from all ethnic backgrounds. The age range of 18–65 years was found appropriate for this scoping review based on CVDs risk factors, such as hypertension, dyslipidemia, and obesity, which often start manifesting early in adulthood. Hence, guidelines on primary prevention of CVDs recommend starting periodic assessments of risk factors every 4–6 years for adults aged 20–39 years [19]. These risk factors for PLWH start to appear at an earlier age compared with the general population, making the age of 18 years a more appropriate. The upper limit of 65 years accounts for the fact that beyond this limit, CVDs risk factors are compounded by other additional comorbidities such as frailty, diabetes, and obesity [20]. Hence, studies show no significant difference in CVD related deaths after the age group 65 years to 75 years between PLHIV and the general population [21].

### Concept

The concept for this scoping review will focus on any literature describing the health education and physical activity interventions for the prevention of CVDs in people living with HIV. More specifically, the scoping review will focus on strategies, components and theories used in these interventions to address CVDs in PLHIV, including outcomes of interest for these interventions. Outcomes of interest will include patient outcomes such as CVDs risk knowledge levels and CVDs risk profiles, focusing on high blood pressure (BP), given its high prevalence as a modifiable risk factor among people living with HIV [22]. Other outcomes to be mapped include body mass index (BMI), lipid profiles, physical activity levels, and overall health and well-being. These outcomes will be mapped to provide a comprehensive understanding, but priority will be given to hypertension related outcomes. Feasibility studies focusing on the acceptability and effectiveness of these interventions will also be included.

### Context

The context in this scoping review will be broad owing to the broad nature of the research question. Literature from an array of settings including hospital settings, community settings, HIV-specific care settings, and non-HIV-specific settings provided they focus on the prevention and care of CVDs in HIV-positive patients will be included. The review will also include studies from diverse geographical regions, and ethnic and sociocultural settings. Encompassing diverse contexts in the scoping review will ensure a comprehensive understanding of health promotion interventions for PLWH.

### Types of evidence sources

The scoping review will include both peer-reviewed and non-peer reviewed publications that are in English language and relevant to the research questions. A variety of sources, including qualitative, quantitative, and mixed methods studies, systematic reviews, editorials, and commentaries focusing on the use of health education and physical activity interventions to address CVDs in PLWH will be included. Grey literature such as policy briefs, government reports, guidelines, dissertations, and relevant conference abstracts will also be included. Experimental studies will include both randomized controlled studies and quasi-experimental studies on interventions for the prevention and control of CVDs in HIV-positive patients. Integrating various research designs and literature sources ensures an exhaustive and proficient view of the topic, enriching both the depth and breadth of insights.

This review will consider literature specifically on health education and physical activity interventions for PLWH, published between January 2000 and June 30^th^, 2025. This time frame was considered to be appropriate because the past two decades has seen a growth in the recognition of the unique cardiovascular risk factors in people living with HIV [23], with subsequent advancement in screening methods and preventive measures. Hence, this time frame allows for a comprehensive exploration of recent developments, advancements in HIV treatment, and a shift towards a more holistic approach, incorporating lifestyle factors like health education and physical activity.

## Methods

The structure and development of this scoping review will apply the framework developed by Arksey and O’Malley [24] and later updated by Peters and colleagues [16] in the JBI methodological guidance while the reporting format will be guided by the PRISMA-ScR extension checklist [25]. The PRISMA-ScR is not a methodological guideline, but a complimentary checklist that will be used together with the JBI methodological guidance [26] to come up with a comprehensive reporting of methods and findings of the scoping review.

Prior to conducting the actual systematic review or scoping review, it is good practice to develop a review protocol as it serves as a road map for the actual review, providing a detailed methodological and analytical approach [27]. We have developed our protocol using the Preferred Reporting Items for Systematic Reviews and Meta-analysis Protocols (PRISMA-P checklist, S1 Appendix) [28]. Should any amendments be made to this protocol during the study, they will be reported in the final manuscript that will report the scoping review results. The date of each amendment will be stated, accompanied by a clear description of the change and the rationale.

### Search strategy

The search strategy to identify eligible studies for this scoping review will be carried out in line with the recommendations from the JBI manual [26]. According to the said Manual, the search strategy will be conducted in three (3) steps namely: Step 1: An initial online search using two search engines (PubMed and Scopus), will be conducted to identify articles relevant to the research question. An analysis of text words and index terms from the titles and abstracts of the retrieved papers will be carried out to develop the full search strategy. Step 2: Using the keywords and Mesh terms identified from Step 1, a second full online search will be applied to PubMed, Scopus, CINAHL, and Embase. Step 3: Manual searches of reference lists for the identified articles will be conducted to identify more eligible articles. In addition to the stated databases, manual search of grey literature such as WHO Digital Publications (Institutional Repository for Information Sharing [IRIS] and Global Index Medicus [GIM]), Google Scholar, and Proquest Thesis and Dissertation Global (PQDT) reports will be conducted to identify relevant literature. The search will also incorporate major clinical trial registries such as the ClinicalTrial.gov, and the World Health Organisation International Clinical Trials Registry Platform (WHO ICTRP), in order to ensure the inclusion of a broad range of evidence including relevant unpublished or ongoing studies. The search will be conducted by the main author with the help of a Librarian who will also assist with the development of the search terms. Peer review of the electronic search strategy used will be conducted by two independent reviewers, who are both supervisors of the main author. In line with the JBI evidence synthesis requirements for a scoping review protocol [29], a full search strategy was applied to PubMed as presented in S2 Appendix of this protocol.

### Study selection

All the retrieved records will be put together and uploaded into EndNote X9 for the removal of duplicates. After the removal of duplicates, the first screening process will be done by two reviewers (MMS and YC), using active learning through the use of an open-source machine learning tool, ASReview, to screen and review articles. ASReview will be used because of its accessibility, time-saving, free, and ready-to-use features [30]. In addition, ASReview improves the efficiency and precision of the screening process, and has been reported to reduce by up to 60% of the workload and serve up to 95% of the screening time compared to manual screening [31]. Because ASReview operates locally, collaboration between the two reviewers will be limited. To address this challenge, each of the two reviewers will install the ASReview locally on their computers and use the same dataset, but apply different prior knowledge and then screen the papers independently until they reach the agreed stopping rule [31].

To get started, each of the two reviewers will upload the file of articles from EndNote into ASReview and then select prior knowledge to train the Active Learning Model based on their background knowledge [32]. Ideally, at least one article is supposed to be labelled relevant and another one irrelevant to offer prior knowledge to ASReview for the initial training of the active learning model [30]. In this review, we will start by labelling about 10 articles as relevant and another 10 articles as irrelevant because it is suggested that selecting more prior knowledge is likely to result in improved efficiency of the active learning process [30].

When using ASReview, the reviewers must decide on when to stop the reviewing process. A good stopping criteria should ensure a balance between the cost of having to label large volumes of articles if the review takes too long and the risk of missing relevant articles if they stop too early [33]. Among the many methods to determine the stopping criterion include the heuristic approaches and statistical approaches [34]. While statistical approaches involve estimating the stopping criteria based on the total number of relevant abstracts in the data set, the heuristic approaches consist of two methods namely time-based and data-driven approaches [34]. The time-based heuristic approach stops after screening a certain percentage of articles while the data-driven approach stops after reviewing a predetermined number (n) of consecutive irrelevant articles in a row [35]. This scoping review will use a combination of the two heuristic approaches because, according to Konig et al. [35], combining stopping rules is likely to enhance the performance of the model.

After reviewing with ASReview, the reviewers will also carry out backward snowballing of reference lists of some relevant articles to mitigate problems associated with machine learning tools such as omission of relevant articles [36]. Backward snowballing will be stopped after evaluating at least 20 articles in a row without finding at least 5 new relevant articles from the reference lists of selected relevant articles [36].

Following the title and abstract screening in ASReview, the two independent reviewers (MMS and YC) will screen the extracted titles and abstracts of eligible articles, followed by a screening of full texts to ensure that only articles that are related to the scoping review objectives are included. A random sample of 25 articles will be done to ensure that the articles are according to the inclusion criteria and the review question [29]. Reviewers will hold discussions on the screened articles to ensure that there is consensus. Should any disagreements arise between the two reviewers, consensus will be achieved by involving a third reviewer (EMN). The articles that do not meet the inclusion criteria will be excluded and reasons for exclusion will be reported accordingly. The selection process will be presented in a flow chart according to the PRISMA-ScR checklist.

It is important to highlight that, unlike systematic reviews, scoping reviews do not need methodological quality evaluations of the included papers, therefore this review will not carry out any methodological assessments for the included papers [25]. However, the process for this scoping review will strictly abide by the JBI methodological guidance and the PRISMA-ScR extension checklist for reporting [25].

### Data extraction

The data extraction process, which in scoping reviews is referred to as ‘data charting’ [18], will be done using the data extraction tool that will be adapted from the JBI standardized data extraction form and will be aligned with the proposed study’s research objectives and questions. To reduce the chance of error and bias, two reviewers (MMS and YC) will be involved in the development of the data extraction tool. The development of the tool will be done iteratively until the reviewers come to a final agreement on the information to be collected. Information to be extracted will include the following as shown in S3 Appendix: Author (s), year of publication, country and setting, aims/purpose, population characteristics, and sample size where applicable, methodology, health education or physical activity intervention strategies and, theoretical frameworks, and evidence of the effectiveness of health education and physical activity interventions in addressing CVDs in PLHIV [37]. Table 1 shows an excerpt of the adapted data charting tool.

**Table 1:** An excerpt from the adapted data charting tool.

Evidence source details and characteristics
**Study details:** Author/s: Year of publication: Title: Journal/Source (Vol, Iss, pg): DOI/URL:
**Context**: Country, settings including geographical, ethnic and sociocultural settings
**Population:** Participant Characteristics such as Age, sex, socioeconomic status, sample size/number)

This scoping review will include all eligible studies regardless of whether they used theories or not. This approach recognizes the fact that despite theory-informed interventions being essential for guiding and evaluating behavior change, many important studies may not explicitly report their theoretical basis. According to Peters and colleagues [38], scoping reviews have a broader approach with the aim of mapping literature and addressing a broader research question. Hence, the proposed approach of including all relevant studies will ensure a comprehensive mapping of the interventions. Data on theories will include whether the study used a theory or not and the name of the theory (E.g. Health Belief model; Social cognitive theory, etc.) if the theory was used.

Data charting will be carried out in an iterative process, whereby the charting table will continually be updated when reviewers come across additional unforeseen data deemed relevant to the review questions.

In addition, a pilot data extraction process will be conducted on at least two to three articles by two members of the research team (MMS and YC) to ensure that all relevant data about this scoping review will be captured [18]. Pilot testing will also ensure that reviewers familiarize themselves with the process and ensure compatibility with the data extraction process. After finalizing the data extraction form, two independent data extractors will conduct the data extraction from each of the included studies/articles. Discrepancies or uncertainties in data extraction will be resolved through discussions or the involvement of a third independent reviewer where the two fail to agree. Following the standards and guidance for conducting scoping reviews, there will be no need for quality assessment for the included articles [16].

### Data analysis

Analysis of the extracted data will mainly be descriptive consisting of quantitative frequency counts of populations, concepts, contexts, and types of studies [39]. In addition, qualitative analysis, using a deductive content analysis, will be employed to analyse data on the strategies, components, and theoretical frameworks used in the included studies. An *a priori* coding framework, which will be developed based on the review’s objectives and research questions, will guide both the data charting and analysis process. This framework will include predefined categories such as strategies and components used in health education and physical activity interventions, theories used, reported outcomes, and any gaps. Two reviewers (MMS and YC) will independently apply the coding framework to a subset of the data to ensure consistency, with discrepancies resolved through discussion or consultation with a third reviewer. Any relevant categories that will emerge during the analysis but may not have been included in the initial framework will also be documented and incorporated where appropriate.

### Ethical statement

This scoping review is part of the PhD project and the protocol for the project has been granted ethical approval (REF: 2023-DEC-018). However, this being a scoping review, ethical approval may not be necessary. Despite this being the case, we may come across articles that may have identifiable information that should be protected. If we come across any potentially identifiable information, strict measures will be taken to ensure confidentiality by not disclosing the source of that information.

### Expected results

The results of the search strategy and process will be presented according to the PRISMA-ScR checklist in the form of a flowchart. A summary of results consisting of information related to the research objectives and questions will be presented in tabular format and diagrams. This will be followed by a narrative description of the contents in the tables or graphs to explain how the results relate to the review questions and objectives as well as any new findings and potential gaps that may be identified.

## Limitations

This scoping review will only include articles published or translated in English. While there are valid reasons for the exclusion criteria, there may be potential biases including language bias, temporal bias, and geographic bias. To mitigate these biases, the review will diversify its search strategy to include relevant grey literature sources and studies from various ethnic, cultural, and regional sources. The review commits to ensuring a comprehensive, transparent synthesis and reporting of the results, together with a thorough consideration of limitations, to enhance the credibility and applicability of its findings, within the specified scope.

## Dissemination plans

The results of the proposed scoping review are intended to inform the design and development of an intervention to prevent CVDs in people living with HIV. The manuscript that will report the scoping review findings will be published in the relevant high-impact peer-reviewed journal. In addition, the findings of the scoping review will be shared with relevant stakeholders through workshops and conferences.

## Supporting information

S1 Appendix PRISMA-P checklist. (DOCX)

S2 Appendix PubMed search query. (DOCX)

S3 Appendix Data charting tool. (DOCX)
