# Supplementary material for: Mapping strategies, components, and theories used in health education and physical activity interventions to prevent cardiovascular diseases in adults living with HIV: A scoping review protocol
Source: PLoS One. 2025 Aug 1;20(8):e0312969. doi: 10.1371/journal.pone.0312969 (PMC12316239; doi:10.1371/journal.pone.0312969)
Supplement: S2 Appendix — (DOCX) [file pone.0312969.s002.docx]

**Appendix S1. PubMed Search strategy (Date: 01/10/2024)**

| **No.** | **PCC** | **Query string** | **Results** | Time |
| --- | --- | --- | --- | --- |
| **#1** | Population | (("People living with HIV"[Title/Abstract] OR "Adults living with HIV"[Title/Abstract] OR "AIDS seropositivity"[Title/Abstract] OR "HIV seroconversion"[Title/Abstract] OR "Human immunodeficiency virus"[Title/Abstract] OR "antiretroviral therapy"[Title/Abstract]) AND ("english"[Language] AND "adult"[MeSH Terms] AND 2000/01/01:2024/12/31[Date - Publication])) AND ((english[Filter]) AND (alladult[Filter])) | 45,645 | 07:22:26 |
| **#2** | Concept 1 | (("Cardiovascular diseases"[Title/Abstract] OR "Myocardial infarction"[Title/Abstract] OR "hypertension"[Title/Abstract] OR "Atherosclerotic disease"[Title/Abstract] OR "Heart disease"[Title/Abstract] OR "stroke"[Title/Abstract] OR "metabolic syndrome"[Title/Abstract] OR "Ischemic heart disease"[Title/Abstract] OR "coronary heart disease"[Title/Abstract] OR "pulmonary hypertension"[Title/Abstract] OR "cardiac events"[Title/Abstract] OR "Adverse cardiac event"[All Fields]) AND "english"[Language] AND "adult"[MeSH Terms] AND 2000/01/01:2024/12/31[Date - Publication]) AND ((english[Filter]) AND (alladult[Filter])) | 321,063 | 07:23:06 |
| **#3** | Concept2 | ("Strategies"[Title/Abstract] OR "Techniques"[Title/Abstract] OR "intervention"[Title/Abstract] OR "theories"[Title/Abstract] OR "health education"[Title/Abstract] OR "Exercises"[Title/Abstract] OR "physical activity"[Title/Abstract] OR "Aerobic exercises"[Title/Abstract] OR "Exercise training"[Title/Abstract] OR "physical fitness"[Title/Abstract] OR "health promotion"[Title/Abstract] OR "Health literacy"[Title/Abstract] OR "health behavior"[Title/Abstract] OR "Community health promotion"[Title/Abstract] OR "Health communication"[Title/Abstract] OR "Health knowledge"[Title/Abstract] OR "healthy lifestyles"[Title/Abstract] OR "patient education"[Title/Abstract] OR "walking program"[All Fields]) AND ((english[Filter]) AND (alladult[Filter]) AND (2000:2024[pdat] | 560,583 | 07:23:40 |
| **#4** | Context | ("hospitals"[MeSH Terms] OR "hospitals"[All Fields] OR "hospital"[All Fields] OR "communities"[All Fields] OR "community"[All Fields]) OR "Community health networks"[All Fields] OR "Health networks"[All Fields] OR "Community Health Centers"[All Fields] OR "fitness center"[All Fields] OR "health facilities"[All Fields] OR "Health resorts"[All Fields] OR "rehabilitation centers"[All Fields] OR "Health clubs"[All Fields] OR "Spar"[All Fields] OR "Wellness center"[All Fields] OR ("fitness centers"[MeSH Terms] OR ("fitness"[All Fields] AND "centers"[All Fields]) OR "fitness centers"[All Fields] OR "gymnasiums"[All Fields]) OR ("fitness centers"[MeSH Terms] OR ("fitness"[All Fields] AND "centers"[All Fields]) OR "fitness centers"[All Fields] OR "gyms"[All Fields])) AND ((english[Filter]) AND (alladult[Filter]) AND (2000:2024[pdat])) | 1,894,312 | 07:24:13 |
| **#5** | PCC | #1 AND #2 AND #3 AND $4 | 111 | 07:25:18 |
