# Supplementary material for: Mapping strategies, components, and theories used in health education and physical activity interventions to prevent cardiovascular diseases in adults living with HIV: A scoping review protocol
Source: PLoS One. 2025 Aug 1;20(8):e0312969. doi: 10.1371/journal.pone.0312969 (PMC12316239; doi:10.1371/journal.pone.0312969)
Supplement: S3 Appendix — (DOCX) [file pone.0312969.s003.docx]

**Appendix S2. Data extraction tool**

| **Evidence source details and characteristics** |  |
| --- | --- |
| **Study details:**  Author/s:  Year of publication:  Title:  Journal/Source (Vol, Iss, pg):  DOI/URL: |  |
| **Context**: Country, settings including geographical, ethnic and sociocultural settings |  |
| **Population:**  Participant Characteristics such as Age, sex, socioeconomic status, sample size/number) |  |
| Key objectives and research questions |  |
| **Study design and methodology**  - Methods  - Study design  - Sample size  - Data collection methods |  |
| **Key findings relating to the scoping review concepts:**  - Health education intervention strategies and components for prevention of CVDs in PLWH  - Physical activity strategies and components used to prevent CVDs in PLWH  - Theoretical frameworks used in HE and PA interventions  - Evidence on the effectiveness of HE and PA interventions in addressing CVDs in PLWH |  |
| Any additional notes |  |
| Name of reviewer: |  |

**Abbreviations:** HE = Health Education; PA = Physical activity; CVDs = Cardiovascular diseases;

PLWH = People living with HIV
